# Micro-sized thin-film solar cells via area-selective electrochemical deposition for concentrator photovoltaics application

**DOI:** 10.1038/s41598-020-71717-0

**Published:** 2020-09-08

**Authors:** Daniel Siopa, Khalil El Hajraoui, Sara Tombolato, Finn Babbe, Alberto Lomuscio, Max H. Wolter, Pedro Anacleto, Kamal Abderrafi, Francis L. Deepak, Sascha Sadewasser, Phillip J. Dale

**Affiliations:** 1 Department of Physics and Materials Science, University of Luxembourg, 4422 Belvaux, Luxembourg; 2 INL - International Iberian Nanotechnology Laboratory, 4715-330 Braga, Portugal

**Keywords:** Chemistry, Energy science and technology, Materials science

## Abstract

Micro-concentrator solar cells enable higher power conversion efficiencies and material savings when compared to large-area non-concentrated solar cells. In this study, we use materials-efficient area-selective electrodeposition of the metallic elements, coupled with selenium reactive annealing, to form Cu(In,Ga)Se_2_ semiconductor absorber layers in patterned microelectrode arrays. This process achieves significant material savings of the low-abundance elements. The resulting copper-poor micro-absorber layers’ composition and homogeneity depend on the deposition charge, where higher charge leads to greater inhomogeneity in the Cu/In ratio and to a patchy presence of a CuIn_5_Se_8_ OVC phase. Photovoltaic devices show open-circuit voltages of up to 525 mV under a concentration factor of 18 ×, which is larger than other reported Cu(In,Ga)Se_2_ micro-solar cells fabricated by materials-efficient methods. Furthermore, a single micro-solar cell device, measured under light concentration, displayed a power conversion efficiency of 5% under a concentration factor of 33 ×. These results show the potential of the presented method to assemble micro-concentrator photovoltaic devices, which operate at higher efficiencies while using light concentration.

## Introduction

Global energy consumption is rising, and photovoltaic modules offer a sustainable source of renewable energy. Currently, the installed capacity is dominated by silicon modules, while strong research efforts have led to significant power conversion efficiency (PCE) improvements of the thin-film technologies based on the absorber layers CdTe and Cu(In,Ga)Se_2_ (CIGSe)^1^. For CIGSe devices the strategies taken to improve the overall PCE include rear contact passivation^2,3^, absorber passivation^4^, using Zn rather than Cd based buffer layers^5^, using the CIGSe device as a sub-cell in a tandem solar cell^6^, and reducing light lost to reflection^7^.

**Figure 1:**
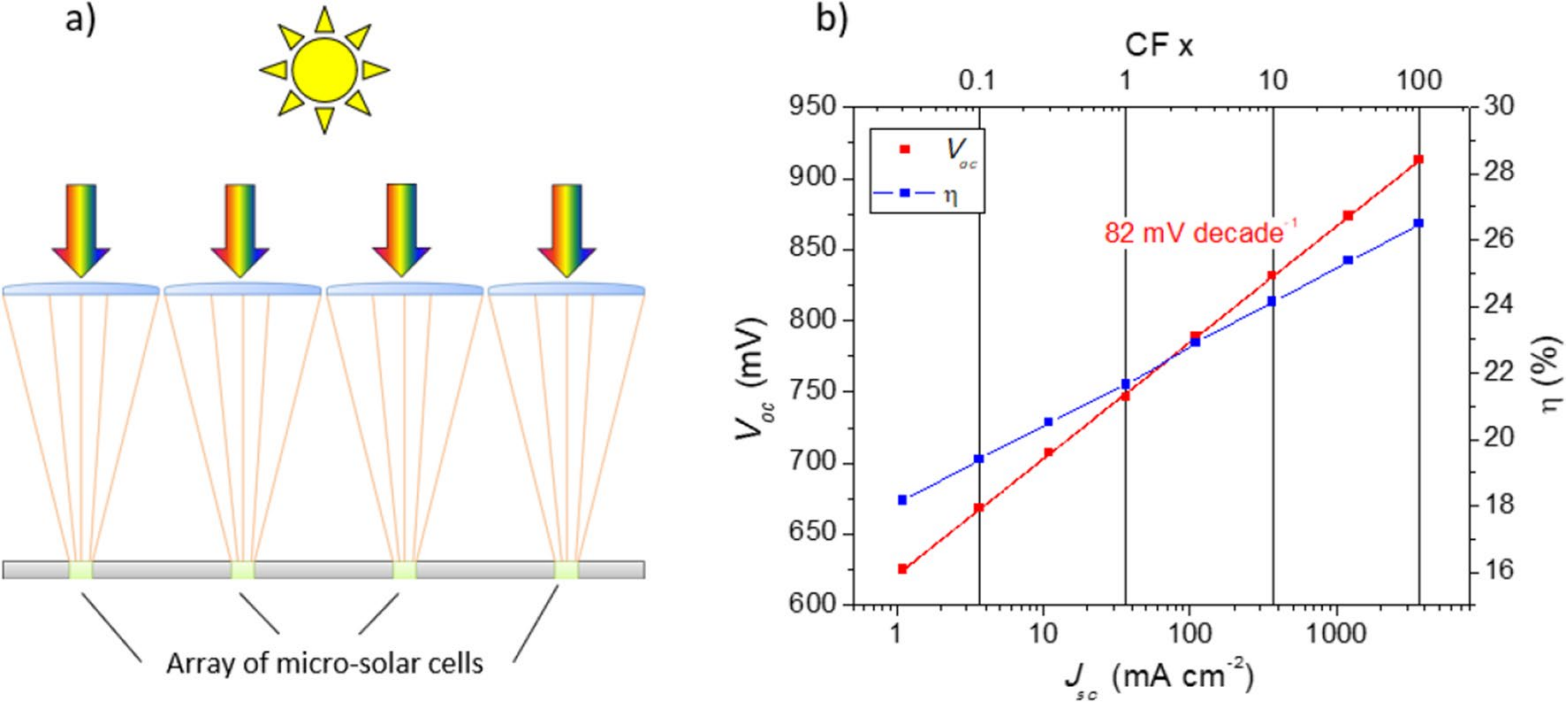
(**a**) Micro-solar cells under concentration. (**b**) Predicted solar cell efficiency (η) and *V*_*oc*_ of a CIGSe solar cell as a function of *J*_*sc*_ and CF based on Eq. (2) using 1 sun parameters obtained by Jackson et al.^8^ and assuming there are no changes in FF, A, and J_0_.

One way to improve the PCE of these technologies even further is to employ concentrated sunlight (Fig. 1a). The PCE (*η*) of a solar cell is defined as the product of the open-circuit voltage (*V*_*oc*_), the short-circuit current density (*J*_*sc*_) and the fill factor (*FF*) divided by the incident light power density (*P*_*in*_):

1 $$\eta =\frac{{V}_{oc}{J}_{sc}FF}{{P}_{in}}$$

An increase in *P*_*in*_ leads to an increase in *J*_*sc*_ proportional to a light concentration factor (CF). The PCE improves under concentrated light because the *V*_*oc*_ increases logarithmically due to its dependence on *J*_*sc*_ according to the rearranged Schockley diode model:

2 $${V}_{oc}=\frac{A{k}_{B}T}{q}\mathit{ln}\left(\frac{{J}_{sc}}{{J}_{0}}+1\right),$$

where *J*_*0*_ is the reverse saturation current density, *k*_*B*_ is Boltzmann’s constant, *A* is the diode factor, *T* is the temperature and *q* is the elementary charge. The changes in PCE and *V*_*oc*_ with increasing illumination intensity are illustrated in Fig. 1b, where we assume that *T*, *J*_*0*_, and *A* are independent of the incident light concentration factor. An additional benefit of light concentration is that less active solar cell surface area is required for harvesting the same amount of light, thus reducing the amount of precious semiconductor needed. The material saving is directly proportional to the light concentration factor^9^, and thus macro (centimetre-sized) solar cell devices can be shrunk to micrometre dimensions by using lenses with high magnifications. Micro-sized devices have the advantage, compared to larger devices, of having a lower series resistance due to the shorter distances carriers need to travel and lower operating temperatures due to the higher surface-to-volume ratio^10–15^. Polycrystalline CIGSe is a suitable material for making micro-sized devices because it achieves high PCEs and the device *V*_*oc*_ is almost insensitive to the surface-area-to-volume ratio, unlike other crystalline systems^16^.

CIGSe is a quaternary compound semiconductor which is normally p-type doped and crystallizes in the chalcopyrite structure with a band gap between 1.0 and 1.7 eV, depending on its Ga content^17^. It has a direct band gap and high absorption coefficient (10^5^ cm^−1^) making it suitable as a solar cell absorber layer^18^. The normal thickness of a CIGSe absorber layer is around 1.5–3 µm which is more than sufficient to absorb all the incoming solar radiation. Recently intensive research is dedicated to thinning this down to 500 nm or less, which is still sufficient to absorb the light but uses four to six times less precious indium and gallium metal^12,13^. One potential drawback of thinning the absorber too much is the increased risk of recombination at the Mo/CIGSe interface which will reduce the devices open circuit voltage. As stated above, another way to save material is to make micro-solar cells where the lateral dimension of the cell is in the micrometer range. A proof-of-concept approach to fabricate micro-concentrator solar cells by Paire et al.^19^, who used co-evaporated CIGSe continuous layers in a standard device stack of Mo/CIGSe/CdS/ZnO/Al:ZnO/Au. A SiO_2_ dielectric layer was inserted between the ZnO and Al:ZnO layers, and individual micro-cells were defined by photolithography. Using this approach, a *V*_*oc*_ of 660 mV at one sun (1 × CF) was increased to 850 mV at 475 × CF. This gain in *V*_*oc*_ with concentration improved the PCE from 16 to 21.3%. It was shown that the lateral miniaturization (5 to 500 µm width) of the micro-cells resulted in negligible spreading resistances, and thus despite the high currents, the *V*_*oc*_ was not impacted due to TCO or back contact losses^19^. However, the *V*_*oc*_ does not increase entirely logarithmically as predicted, but with a slight downward curvature at high light concentrations, from which the authors concluded that the internal resistivity of the CIGSe absorber layer was sufficiently high to cause a voltage drop at high light concentrations^20^. Other top-down approaches to make micro-cells, starting from co-evaporated large-area layers, have also achieved PCE above 20% under concentrated illumination^21–23^. CIGSe micro-cells were also realized from electrodeposited (ED) absorber layers similarly to the proof-of-principle devices described above^24^. The record micro-cell had a PCE of 15% under 33 × CF^9^, a significant increase from 5% at 1 × CF illumination.

The above approaches are an excellent proof-of-concept, but do not achieve materials savings since they all start from large continuous layers. Several bottom-up preparation strategies that reduce consumption of critical raw materials were achieved using laser nucleation and LIFT techniques, reaching PCEs of 3.4% at 20 suns^23,25–27^. Duchatelet et al.^24^ reported the fabrication of CIGSe stripes with sub-millimetre width using area-selective electrodeposition (ASED). The deposits were restricted in size by virtue that the Mo back contact consisted of line arrays on a bare glass substrate rather than the normal continuous layers covering the glass. The authors achieved a PCE of 5.3% for the 1 cm long 105 µm wide lines, although they mention the CIGSe annealing process could still be improved since they recognize that temperature gradients caused by cell geometry and the unwanted reaction of selenium on the glass likely forming volatile sodium selenide species was detrimental for high quality absorber layers^28^. The line array design is an excellent way to achieve semi-transparent photovoltaics, but upscaling the electrodeposition to larger sized areas might prove difficult, since there will be considerable voltage drop along each line due to increased resistance of trying to pass current through a narrow line rather that a sheet. Sadewasser et al.^10^ demonstrated an alternative ASED approach: CuInSe_2_ (CISe) was electrodeposited from a single deposition bath containing Cu, In, and Se in holes that were etched into an insulating SiO_2_ layer on top of a continuous Mo substrate and reported a device array with a *V*_*oc*_ of 94 mV at 1 sun. A literature summary of the solar cell parameters obtained for different micro-devices prepared via bottom-up approaches is found in Table 1.

**Table 1:** Summary of solar sell parameters obtained for micro-devices prepared via bottom-up approaches.

References	Absorber	Illum.(CF)	*V*_*oc*_ (mV)	*J*_*sc*_ (mA cm^−2^)	η (%)
Duchatelet et al.^18^	Electrodeposition	1 ×	368	30.2	5.38
Sadewasser et al.^3^	Electrodeposition	1 ×	94	–	0.26
Heidmann et al.^20^	LIFT	1 ×	145	≈ 4	1.4
Ringleb et al.^19^	Laser nucleation	3 ×	≈ 370	–	3.1
Present work	Electrodeposition	18 ×	525	447	5.2

Concentration factor (CF) is defined as ratio between the *J*_*sc*_ obtained under light concentration and the *J*_*sc*_ obtained under AM1.5 1 sun illumination.

Here, we present amodified bottom-up ASED approach to forming micro-CIGSe absorber layers, where instead of depositing all the semiconductor elements at once, we deposit the metal elements in two steps, and introduce the chalcogen by reactive annealing. This approach of reactive annealing of electrodeposited metal stacks already led to a PCE of 17.3% for large area CIGSe^26^, which is significantly higher than the 9% reported for the single deposition bath approach^27^. We investigate if the stacked layer approach also translates into higher efficiencies in the micro format. Therefore, we electrodeposited a Cu layer from an aqueous solution, and then an (In + Ga) layer from an ionic liquid into a patterned insulating SiO_2_ layer deposited on top of a Mo back contact. An array of recessed circular areas with a diameter of 200 µm exposed the Mo to the electrolyte during electrodeposition to enable the deposition of metal layer islands. The advantage of using the SiO_2_ layer is that it protects the underlying Mo layer from being selenized during the annealing process hopefully avoiding increases in the solar cell series resistance due to the formation of MoSe_2_. We investigate two different absorber thicknesses, one around 0.5 µm to see if it is possible to reduce the quantity of CIGSe in the device to the bare minimum, and one of around 1.5 µm to act as a comparison to the normal absorber layer thicknesses of large area films. After reactive annealing, the resulting absorber layers were characterized to determine their composition, phase, morphology, and band gap. Finally, the current–voltage properties of two solar cells formed from the two absorber layers with different thicknesses are investigated over three orders of magnitude light concentration. The thicker device showed an open-circuit voltage of 525 mV under 18 × concentration, which is the highest reported value for any material efficient bottom-up deposition approach for CIGSe.

## Methods

### Fabrication of SiO_2_ template patterns

**Figure 2:**
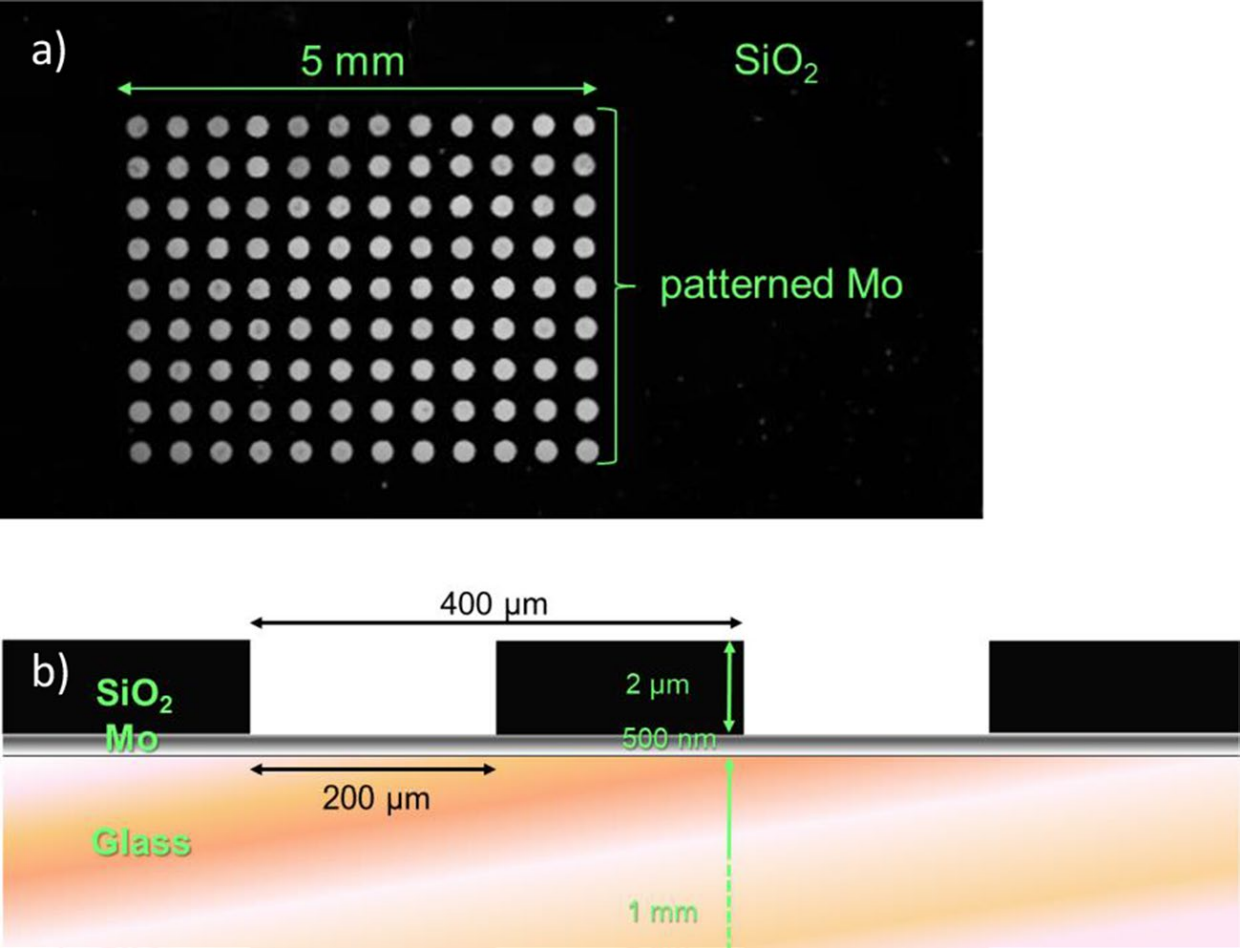
Overview of SLG/Mo sample patterned with SiO_2_: (**a**) top-view optical microscope image and (**b**) schematic representation of the profile view of the pre-structured substrates.

Micro-patterned substrates were prepared according to the procedure described by Sadewasser et al.^10^ In brief, 1 mm thick soda lime glass (SLG) is covered by a 500 nm Mo layer deposited by sputtering and after that, a 2 μm thick SiO_2_ layer deposited by plasma enhanced chemical vapour deposition. Direct-write laser lithography is used to define the desired hole pattern. The SiO_2_ is then etched down to the Mo layer by reactive ion etching and residuals are removed by plasma etching. An overview of the resulting pattern and a representation of its cross-section are depicted in Fig. 2.

### Absorber preparation and characterization

Cu(In,Ga)Se_2_ is grown by electrodeposition of the metals and subsequent reactive annealing in a Se-containing atmosphere^29^. The metal electrodeposition is split into two: first the deposition of Cu from an aqueous bath and second the co-deposition of In and Ga from a deep eutectic solvent. The Cu plating bath is prepared by dissolving CuSO_4_·7H_2_O (99.99%, Alfa Aesar), NaOH (semiconductor grade, Sigma-Aldrich), sorbitol (99%, Sigma-Aldrich) in ultra-pure water, in order to obtain a dark blue solution with the concentrations of 3.0, 0.2, and 0.1 M, respectively. Prior to deposition, 70 µl of Empigen was added to the solution at room temperature. The substrates were placed onto a rotating disk electrode (RDE) as a working electrode with a Ag/AgCl electrode used as reference and a Pt wire used as counter electrode. The potentiostatic electrodeposition of Cu was carried out at room temperature (E_dep_ =  − 1.15 V vs Ag/AgCl), with a RDE speed of 200 RPM. The deposition is stopped when the desired cut-off charge density of is reached. The substrate is subsequently washed, dried, and stored under nitrogen until the next deposition step.

In and Ga are electrodeposited on top of the Cu layer inside a nitrogen-filled glove box. The deep eutectic solvent for the plating consists of urea (99%, Sigma-Aldrich), and choline chloride (> 98%, Sigma-Aldrich), mixed in a (1:2 molar ratio) at 60 °C. InCl_3_ (anhydrous 99.999%, Alfa Aesar) and GaCl_3_ (ultra-dry 99.999%, Alfa Aesar) are then dissolved in the deep eutectic solvent at the same temperature, in order to obtain concentrations of 50 mM and 25 mM, respectively. For this deposition, a Ag pseudo reference electrode is used, whilst the other electrodes remain the same as previously. The electrodeposition is carried out at 60 °C (E_dep_ =  − 1.2 V vs Ag), with a RDE speed of 300 RPM. Charge density cut-offs and targeted stoichiometry are described in Table 2 for the different thickness samples. The resulting metallic micro-deposits are then washed multiple times in air with absolute ethanol and deionized water. From here on, the two samples are labelled S500 and S1500 respectively, where the number represents the thickness of the absorber layer, as expected from the deposition charges (see Table 2).

**Table 2:** Electrodeposition parameters used to obtain the metallic precursors.

Sample	Cu dep. charge density (C cm^−2^)	In + Ga dep. charge density (C cm^−2^)	Target absorber thickness (µm)
S500	0.33	0.45	≈ 0.5
S1500	0.94	1.28	≈ 1.5

Following previously published procedures for large area electrodes (≈ 4 cm^2^)^24^, the following metal ratios should be observed due to the different faradaic plating efficiencies—[n(Cu)]/[n(In + Ga)] = 0.9 and [n(Ga)]/[n(In + Ga)] = 0.3.

The Cu-In-Ga micro-deposits, S500 and S1500, are converted into Cu(In_1−x_Ga_x_)Se_2_ via reactive annealing using a rapid thermal processor. In the presence of 80 mg of Se (99.999%, Alfa Aesar) they are first kept at 100 °C under a roughing pump vacuum for 30 min, then conditioned with 10 mbar of forming gas (5% H_2_/N_2_) and heated at a rate of 30 °C s^−1^ up to 450 °C. After 20 min annealing, the system is left to cool down to room temperature.

Top-view scanning electron microscopy (SEM) micrographs were taken with a Hitachi SU-70 and energy dispersive X-ray spectroscopy (EDX) measurements were collected with an Oxford INCA X-MAX analyser coupled to the SEM. The lamellas used for qualitative EDX analysis were prepared using a dual beam Focus Ion Beam Scanning Electron Microscopy (FIB-SEM) equipped with a STEM detector at 30 kV. Photoluminesence spectra of the absorber layers were measured with a home-built setup using a 660 nm laser excitation, a grating monochromator and an InGaAs detector array. Raman spectroscopy was performed using a 633 nm laser excitation in combination with a 2,400 lines/mm grating with a Renishaw inVia micro-Raman spectrometer.

### Device preparation and characterization

The prepared micro-absorbers were etched in a 5 wt% KCN aqueous solution for 30 s, after which CdS was deposited on top via chemical bath deposition^29^. Intrinsic ZnO and Al-doped ZnO were deposited via magnetron sputtering^30^ and individual 200 µm diameter holes were electrically isolated by mechanical scribing.

Current–voltage (JV) curves were either measured under AM1.5 illumination using a AAA class solar simulator or at various light concentrations (spanning from 0.04 × to 78 × CF) under a 660 nm continuous wave laser. Shunt resistance (R_shunt_) and series resistance (R_series_) were extracted following the Hegedus-Shafarman analysis^31^. External quantum efficiency (EQE) measurements were performed in a home-built setup calibrated with silicon and InGaAs photodiodes. All measurements were recorded at room temperature.

## Results

### Absorber formation and morphology

**Figure 3:**
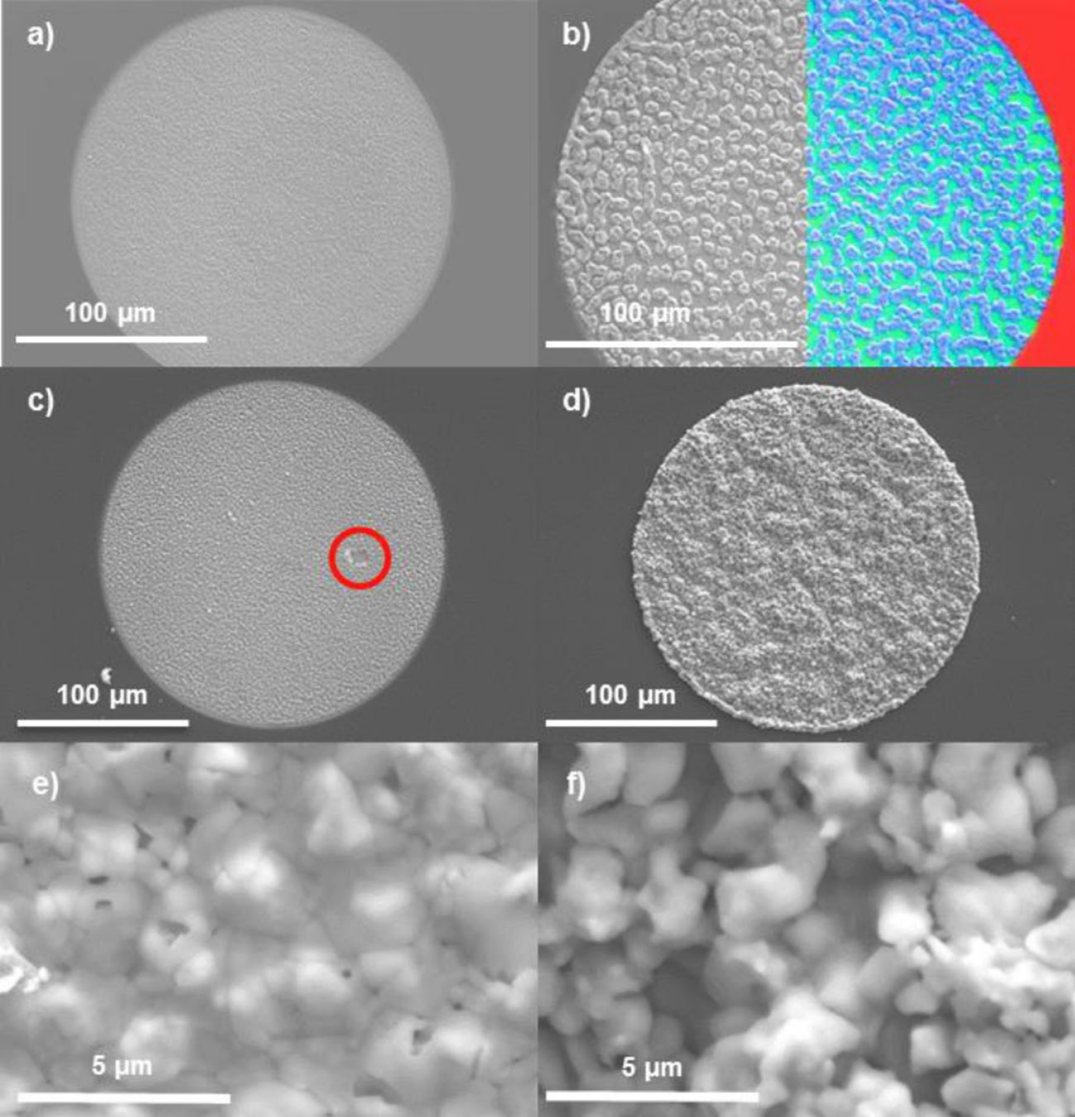
SEM micrographs of micro-deposits: (**a**) Cu electrodeposited onto Mo with 0.33 C cm^−2^ (**b**) In-Ga electrodeposited onto Cu using 0.45 C cm^−2^ (EDX generated image shows Si in red, Cu in green and In in blue), (**c**,**e**) S500 after reactive annealing with Se, (**d**,**f**) S1500 after reactive annealing with Se.

Figure 3a shows a micrograph of the electrodeposited Cu in a 200 µm diameter hole in the SiO_2_ layer. The Cu layer appears smooth and continuous throughout the hole, and the SiO_2_ layer appears undamaged by the electrodeposition process. Figure 3b shows a micrograph of the subsequently electrodeposited In-Ga layer with an EDX elemental map over-lay on half the micrograph showing Si in red, Cu in green and In in blue. Here, 5–10 µm wide In islands are uniformly distributed on the surface of the Cu layer. Figure 1 of the supplementary information shows a cross-section of both S500 and S1500 before selenization. In both cases a continuous Cu-In-Ga layer is observed on top of the Mo, which stops at the edge of the SiO_2_ template.

Figure 3c shows the CIGSe micro-absorber layer S500 prepared from the Cu–In–Ga precursor after reactive annealing in a Se-rich atmosphere. This layer appears smooth and covers the whole surface except for a small region indicated by the red circle. Absorber S1500 appears rougher (Fig. 3d) than S500 but covers the entire surface of the hole. For both samples, the high magnification micrographs reveal pinholes in the absorber layer and apparent grain sizes on the order of 1–2 µm (Fig. 3e,f).

### Absorber characterization

In the following section the absorber composition, the phases present, and the band gap are probed by EDX, Raman and photoluminescence spectroscopies. Raman spectroscopy is preferred over X-ray diffraction to confirm the presence of the chalcogenide Cu(In,Ga)Se_2_ due to the more localized nature of the measurement technique.

A summary of the elemental ratios CGI (= [Cu]/[Ga] + [In]) and GGI (= [Ga]/[Ga] + [In]) determined by EDX is shown in Table 3. The underlying atomic percentage values are given in the supporting information (Table S1). Here, the analysis software assumes that the elements are distributed uniformly throughout the depth of analysis, although this is unlikely to be the case since Ga often segregates towards the back of the film, as is observed for CIGSe prepared from metal stacks^32^. S500 shows a CGI ratio of 0.93, similar to what was aimed at from the cut-off depositions charges used. Contrarily, S1500 shows a lower than expected CGI, indicating the formation of a Cu-poor absorber, most likely due to an over deposition of In. For S500 a GGI of 0.12 was measured, lower than the target value of 0.30 (using the cut-off charge densities ratios and the [InCl_3_]/[GaCl_3_] ratio optimized for larger area depositions as shown in Malaquias et al.^29^). Surprisingly, for S1500, no measurable Ga content was found (the EDX detection limit is around 1%). This result indicates a dependence of the composition of the absorber on the electrodeposition conditions. Furthermore, it points to a change in deposition behaviour when using microelectrodes compared to (large) centimetre sized electrodes, most likely due to an added diffusion effect from the formation of hemispherical diffusion layers in said microelectrodes^33–35^.

**Table 3:** CGI and GGI ratios obtained from EDX measurements.

Absorber	CGI	GGI
S500	0.93 ± 0.16	0.12 ± 0.06
S1500	0.64 ± 0.08	–*

EDX was measured in a 100 µm square area inside the micro-absorber.

*Determined GGI for S1500 was within the margin of error.

**Figure 4:**
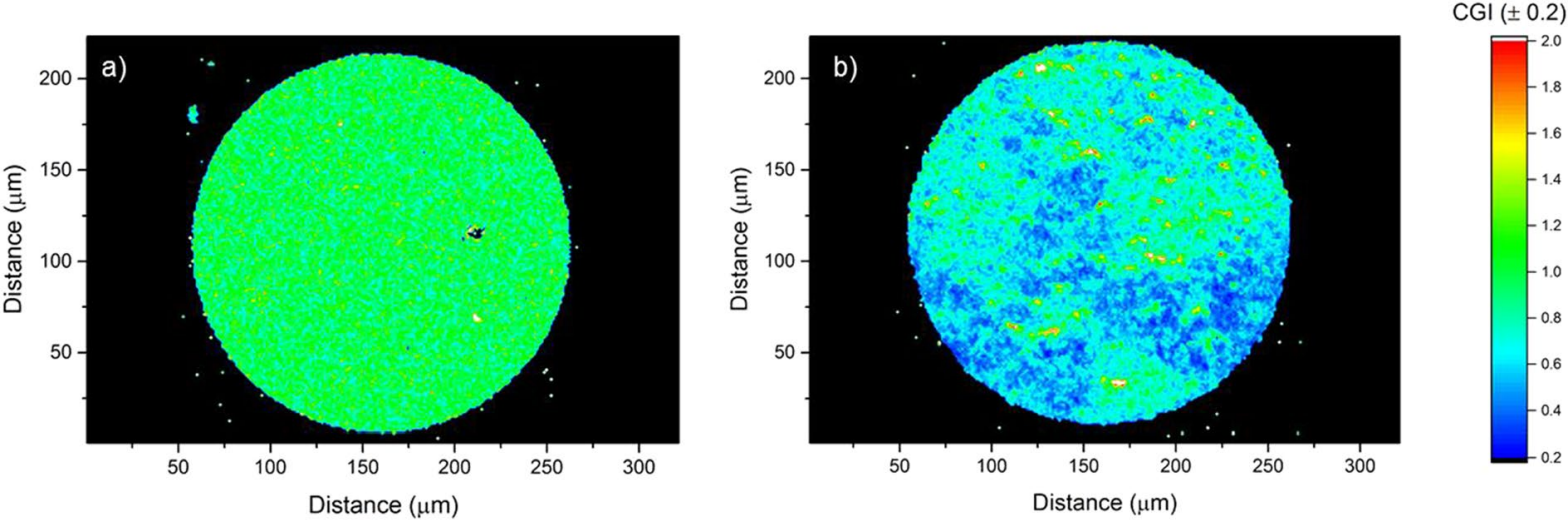
Contour plots of apparent CGI ratio across the micro-absorber (**a**) S500 and (**b**) S1500 obtained from EDX mapping data.

In order to explore the lateral homogeneity of the absorber layers, the top-view EDX composition is mapped with 1 µm resolution and the resulting CGI ratios are shown as contour plots (Fig. 4). S500 has a relatively uniform composition with no obvious chemical gradients or large-scale inhomogeneity. The film is mostly in the stoichiometric range with some randomly scattered areas of a few microns in size with CGI values between 0.6 and 0.8, and a handful of micron-sized spots that are copper rich with CGI values around 1.5. For S1500, the absorber layer is macroscopically inhomogeneous with large areas that are extremely Cu poor with CGI ratios from 0.3 to 0.7, and other large areas near stoichiometry. Like S500, there are also a few Cu-rich spots. We hypothesize that the lateral variations in CGI observed for S1500 are because In islands nucleated on the Cu layer with lower density during the In/Ga electrodeposition process, leading to an over deposition of In in certain areas compared to other areas. This might also explain the higher roughness observed for S1500 (Fig. 3d) since taller In islands would lead to a thicker absorber layer growing out of the island during reactive annealing.

**Figure 5:**
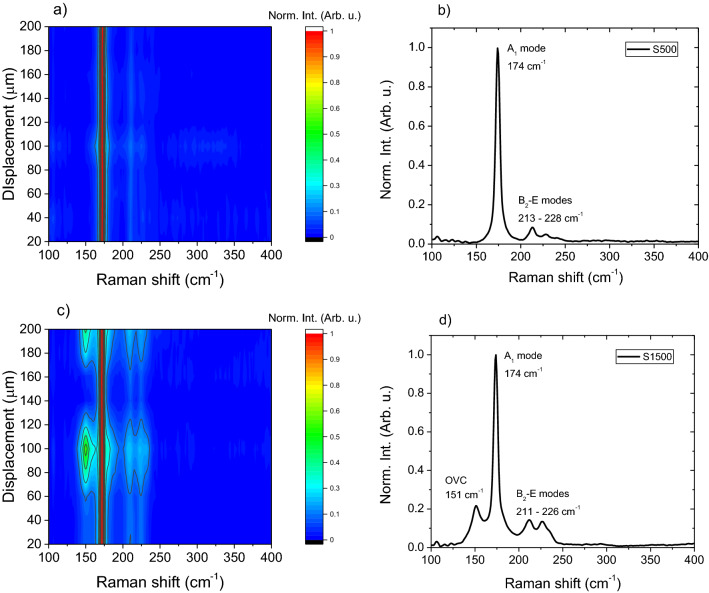
Raman contour plots and corresponding average spectrum taken from the surface of the absorber for S500 (**a**,**b**) and S1500 (**c**,**d**). Point measurements were taken in a line across the surface of the micro-absorber with a 20 µm spacing and an excitation wavelength of 633 nm.

Figure 5 displays the Raman spectroscopy analysis of the prepared absorbers. Cu(In_1−x_Ga_x_)Se_2_ shows a dominant peak at 174 cm^−1^ for CuInSe_2_ and at 184 cm^−1^ for CuGaSe_2_, corresponding to the main vibration mode A_1_ (vibration of Se while Cu, In/Ga remain at rest)^36^. Point measurements are taken across a line over the surface of the absorber samples with a 20 µm spacing (Supplementary Information Fig. S3) and the resulting spectra are plotted against the measurement displacement in contour plots (Fig. 5a,c). The average spectrum is plotted in Fig. 5b,d. For S500, an intense peak at 174 cm^−1^ is observed for all measurements, as indicated by the red vertical line in the contour plot. For S1500 the previously described mode is observed, with an additional mode at 151 cm^−1^ observed in some spots, attributed to the In rich ordered vacancy compound (OVC) CuIn_5_Se_8_^37,38^. The occurrence of the OVC in some spots, as shown by the contour plot, agrees well with the very low Cu content on several spots measured by spatially resolved EDX (Fig. 4). Low intensity modes in the range of 211–228 cm^−1^ are also observed for both samples and are attributed to B_2_—E modes of CIGSe^36^. These modes are more sensitive to the GGI ratio than the A_1_ mode. A shift of the B_2_—E modes of S500 to higher wavenumbers (213–228 cm^−1^) when compared to S1500 (211–226 cm^−1^) indicates the presence of Ga in S500, as predicted by the GGI determined from EDX measurements^39^.

**Figure 6:**
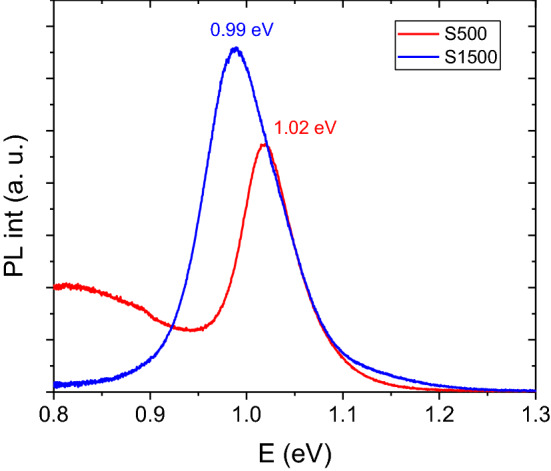
Photoluminescence spectra taken at room temperature of the prepared micro-absorbers.

Photoluminescence spectra measured with a 660 nm laser excitation for both samples are presented in Fig. 6. Cu(In_1−x_Ga_x_)Se_2_ has a band gap around 1.0 eV for CuInSe_2_ and 1.7 eV for CuGaSe_2_, with band gap values varying almost linearly with composition^17^. For S500, a peak at 1.02 eV is observed, corresponding to the band-to-band recombination at room temperature, which gives an approximation of the band gap (E_g_). A band gap value of 1.02 eV for a slightly Cu poor absorber layer with CGI of 0.93 likely indicates the presence of a small amount of Ga, in agreement with the EDX measurements, since a slightly Cu-poor absorber without Ga has an expected band gap of around 0.99 eV^40^. However, a broad sub band gap peak around 0.8 eV is also observed, indicating the presence of a deep level defect. Previously, the presence of such a peak has been observed in Cu rich absorber layers^41^, although this may also indicate impurities from the electrodeposition process. For S1500 a single peak centred at 0.99 eV is observed, corresponding to the main band-to-band transition. This lower band gap corresponds well to a Cu-poor sample without any Ga^40^, in agreement with the EDX measurements. To summarize the PL measurements, S1500 appears to be purely CuInSe_2_ whilst S500 likely contains a small amount of Ga and shows the presence of a deep defect.

### Cross-sectional analysis

**Figure 7:**
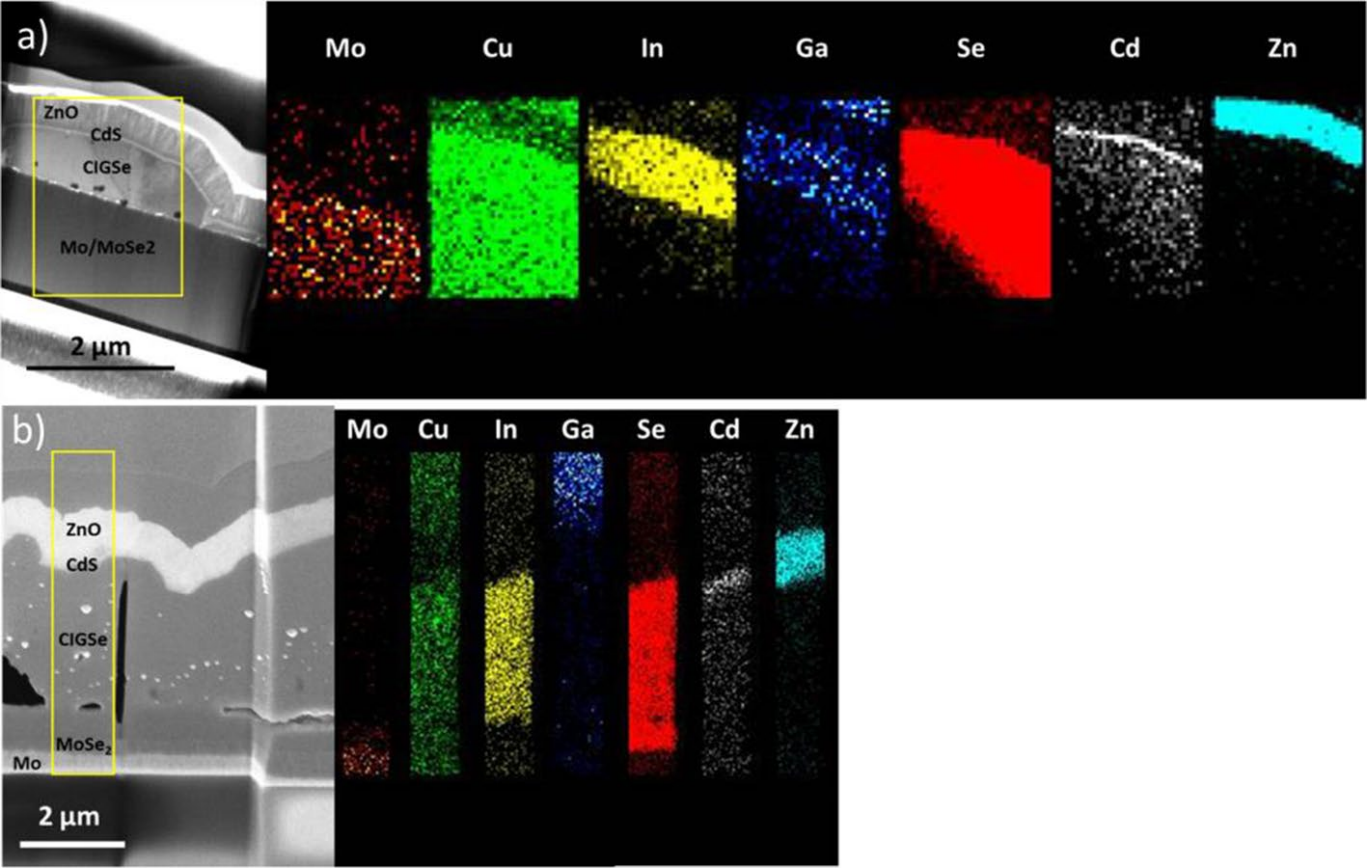
FIB-SEM lamella micrographs and elemental composition for devices prepared using micro-absorbers. (**a**) Bright-field image of S500 and (**b**) high-angle annular dark field (HADDF) image of S1500 with their corresponding EDX maps. Compositional analyses were done in the areas where the largest thickness was observed.

To investigate the absorber thickness and depth-dependent qualitative composition, cross-sectional slices of finished devices were prepared by FIB-SEM. Figure 7 displays FIB lamella micrographs and qualitative elemental mapping profiles by EDX. The stacked architecture of the devices Mo/Absorber/CdS/ZnO can be observed in both samples. For S500 (Fig. 7a) the thickness of the absorber layer fluctuates between 100 and 900 nm. S1500 (Fig. 7b) is thicker in the range 2 μm, as expected from the higher cut-off charges used during deposition. Due to the destructive lamella preparation process, some Cu bright inclusions and voids can be observed in the absorber layers. Additionally, the Cu signal is present in all layers for both samples, due to contamination caused by the copper grid used as support for the sample. The deposited CdS and ZnO window layers are conformal with estimated thicknesses of 50 nm and 500 nm, respectively. Some Cd appears to have inter-diffused into the absorber layer during cell processing which has previously been observed^42^.

For the device prepared with S500 (Fig. 7a), the In map shows an apparent homogeneous distribution in the absorber layer. The Se map shows its presence not only in the absorber layer but clearly extending into the Mo bottom layer, indicating the formation of an interfacial MoSe_2_ layer during the reactive annealing step, despite the relatively low annealing temperature of 450 °C. Ga is weakly detected in the absorber layer, again in agreement with the measured top view EDX (Table 1). The qualitative cross-sectional localized measurement appears to show Ga spread equally through the depth of the absorber layer. However, a Ga-ion beam is used for the milling process that creates the lamellas observed, causing contamination of this element in other layers and hinders an accurate estimation of the distribution of this element over the depth of the absorber. Its contaminating presence can also be observed in the ZnO layer. For the device prepared with S1500 (Fig. 7b), a similar description of the elemental profiles is observed. The main difference lies in the lack of measurable Ga signal in the area of the absorber layer, as expected from the GGI measured from the top view EDX. The Se signal seems to extend both into the Mo bottom layer and partially to the top CdS layer, indicating a diffusion of this element into both layers. To summarize the cross-sectional measurements, both samples show the presence of a MoSe_2_ layer, with S500 showing a small amount of Ga and being significantly thinner than S1500.

### Device optoelectronic characterization

**Figure 8:**
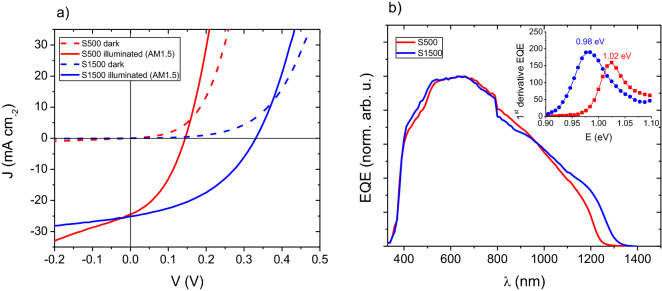
(**a**) JV curves measured in the dark and under one sun (AM1.5) illumination and (**b**) EQE spectra of the micro-solar cells. EQE spectra have been normalized to each respective maximum. Inset in (**b**) shows band gap estimation from the 1st derivative of EQE with respect to energy.

To initially assess the micro-solar cells, current–voltage (JV) curves and EQE spectra were measured (Fig. 8) and a summary of the solar cell parameters extracted by the Hegedus-Shafarman method^31^ is given in Table 4. Devices made from the absorbers S500 and S1500 have power conversion efficiencies of 1.4 and 3.7%, and open-circuit voltages of 144 and 331 mV, respectively, at 1 sun (AM1.5) illumination. The *V*_*oc*_ are low compared to state-of-the-art low bandgap CIGSe devices which achieve 577 mV^43^. There are several reasons for this. For S500 the low *V*_*oc*_ could be attributed to back surface recombination since there is a high recombination velocity at the CIGSe/Mo interface and electron hole pairs would be generated near this interface. Indeed, the thicker S1500, where electron hole pairs would be generated far from the back interface, does have a higher *V*_*oc*_ than S500. However, a normal large area CIGSe solar cell made using similar conditions as S1500 had a *V*_*oc*_ of 427 mV. The key problem appears to be that the micro-solar cells suffer from low shunt resistances, R_shunt_, of 24 and 97 Ω cm^2^ for S500 and S1500, respectively. For efficient devices, R_shunt_ is typically one order of magnitude larger than measured here. A low R_shunt_ is caused by shorting between the back contact and the front contact which may be due to pinholes or conductive secondary phases in the absorber layer itself, a too thin absorber layer, or delamination of the SiO_2_ interface at the CIGSe interface. As shown in SI Fig. 2 we discount delamination at the SiO_2_ interface as the cause of a low R_shunt_, since the cross section of both samples clearly shows the interface to be intact, and the absorber layer clearly separates the conducting layers. Most likely, the low R_shunt_ is caused by the presence of pinholes (Fig. 3e,f), and the presence of Cu-rich areas which are known to be conductive (Fig. 4). These pinholes and secondary phases were not observed in previous work using the same electrodeposition methodology on large area substrates^29^.

**Table 4:** JV parameters extracted for the devices prepared with the electrodeposited absorber layers.

	*V*_*oc*_ (mV)	*J*_*sc*_ (mA cm^−2^)	FF	η (%)	R_shunt_ (Ω cm^2^)	R_series_ (Ω cm^2^)
S500	144	24.5	0.39	1.4	24 (282)	0.98 (0.5)
S1500	331	25.1	0.42	3.5	97 (3,495)	0.70 (1.3)

R_shunt_ and R_series_ values were extracted from both light and dark JV curves (in parenthesis).

Nonetheless, the *V*_*oc*_ for both devices reported here is significantly higher than the previously reported 94 mV for a CISe device using a similar SiO_2_ template^10^. Indeed as hypothesized in the introduction, as for large area solar cells, the electrodeposition route of stacked metal precursor layers leads to better opto-electronic properties compared to the deposition of all elements simultaneously. Surprisingly, the short-circuit current (*J*_*sc*_) for both devices is quite similar, despite the differences in their thickness, which suggests current collection is not improved by increasing thickness of the absorber layer.

To investigate current collection efficiency, the relative external quantum efficiency (EQE) of the devices was measured (Fig. 8b). EQE spectra are discussed relatively and presented with arbitrary units normalized to the respective maximum because the probe beam size was on the order of the size of the device, so we cannot confidently assume that all photons hit the absorber area. For both spectra, a sudden break at 800 nm can be observed due to the switching of a light source in the setup which caused a slight deviation of the beam position. The spectra show some parasitic absorption from the CdS window layer between 400 and 580 nm and reach their maximum at around 600 nm. For higher wavelengths, a continuous drop in the EQE response is observed. The poor carrier collection at long wavelengths is indicative of a short minority carrier transport length. Additionally, long wavelength losses for S500 could be attributed to an incomplete absorption since its thickness is well below a micron in some parts (Fig. 7a). The band gap values are estimated from the maximum of the first derivative of the EQE spectra at long wavelengths. The obtained values of 1.02 and 0.98 eV for S500 and S1500, respectively, fit very well with the peak maxima in the PL spectra (Fig. 6).

### Device light concentration series

**Figure 9:**
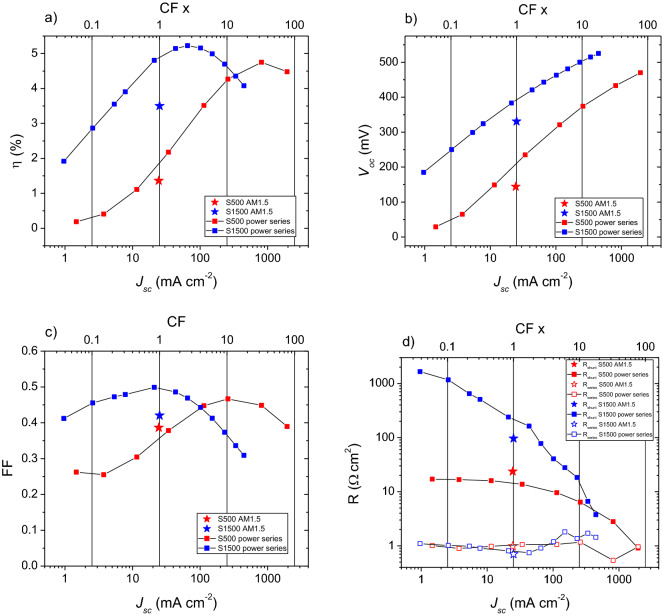
Variation of JV parameters with excitation intensity under 660 nm illumination. (**a**) Efficiency, (**b**) *V*_*oc*_, (**c**) FF, (**d**) R_shunt_ and R_series_.

To study the photovoltaic performance under concentrated light, the devices were illuminated with a 660 nm laser over three orders of magnitude of intensity. According to Eq. (2), there should be a logarithmic increase of the *V*_*oc*_ with illumination intensity, leading to an increase in efficiency. A concentration factor (CF) representing light intensity was defined as a ratio between the *J*_*sc*_ obtained in the power series and the *J*_*sc*_ obtained in the solar simulator (calibrated for 1 sun)^44^. The solar cell parameters η, *V*_*oc*_, FF, R_shunt_, R_series_, and their dependence on illumination intensity are plotted (Fig. 9). For comparison, the data points from the AM1.5 solar simulator JV curves are added.

As expected from Eq. (2), starting from below 0.1 × CF an initial increase in efficiency for both devices is observed with increasing illumination intensity, mainly due to the increase in *V*_*oc*_ and in FF (Fig. 9a–c). Maximum PCE of 4.7% at 33 × CF for S500 and 5.2% at 3 × CF for S1500 are reached. This means there is an increase in efficiency for both devices when comparing with the values obtained with AM1.5 in the solar simulator of 1.4 and 3.5%, respectively. For higher illumination intensities, a decrease in efficiency is observed that can be explained by a combination of the decrease of both FF (Fig. 9c) and R_shunt_ (Fig. 9d). For the intensity range explored, *V*_*oc*_ continuously increases, with a slightly reduced slope at higher illumination intensities, similar to the observations by Paire et al.^19^ for co-evaporated CIGSe micro-cells. On the other hand, Heidmann et al.^45^ and Ringleb et al.^46^ reported a decrease of *V*_*oc*_ at around 30–40 suns for CISe micro-solar cells prepared via bottom-up laser nucleation and LIFT techniques. In our case, an average rate of increase of around 127 and 150 mV dec^−1^ for S500 and S1500, respectively, is determined. This is nearly twice as high as the 82 mV dec^−1^ predicted in Fig. 1 for a record CIGSe solar cell with a diode factor (A) of 1.38. Our results indicate A = 2.2 (S500) and A = 2.5 (S1500), which is too high for efficient solar cells. Additionally, the measured *V*_*oc*_ under AM1.5 illumination is lower than the one measured with 660 nm light which will be discussed in more detail below. Overall, a maximum *V*_*oc*_ of 525 mV for S1500 was achieved under a concentration factor of 18 × , which is the highest reported value for micro-cells prepared via bottom-up approaches and under light concentration like the one presented in this work.

The R_shunt_ obtained for the devices measured under the 660 nm laser and the AM1.5 illumination show different values. This means there is an apparent dependence of R_shunt_ on the illumination intensity and wavelength. For S500, a slightly larger R_shunt_ value of 24 Ω cm^2^ is determined from the AM1.5 curve when compared to the closest value in the power series (14 Ω cm^2^) and a higher FF value is observed. For S1500 however, a lower R_shunt_ value for the AM1.5 curve is found with a corresponding lower FF value. This implies a strong dependence of the FF on the R_shunt_ which is expected and well described by Green^47^.

Interestingly, for the device prepared with absorber S500, which showed lower estimated thickness and more evidence of pinholes, R_shunt_ decreases by one order of magnitude over the three orders of magnitude of illumination probed compared to three orders of magnitude change for S1500. This means that the device prepared with absorber S1500 reaches a peak efficiency at a lower concentration factor than S500, due to its steeper decrease of R_shunt_ with illumination intensity (Fig. 9d). On the other hand, any possible temperature increase from the increasing illumination intensity does not appear to have a strong dependence on R_series_, as it is stable over the range studied (Fig. 9d), as predicted by Sadewasser et al.^11^.

**Figure 10:**
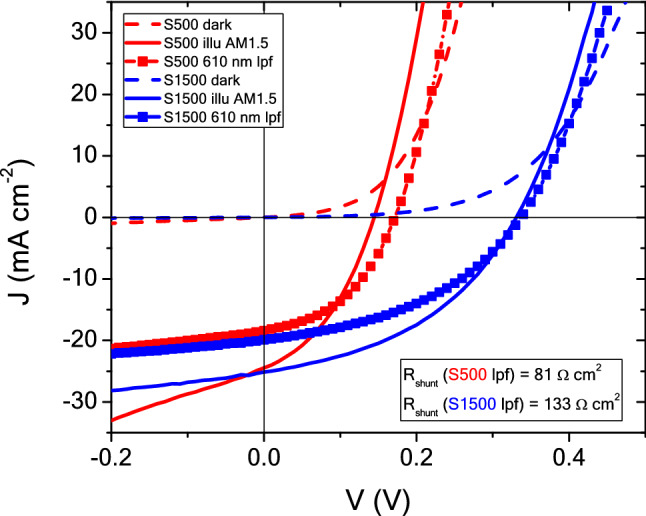
JV curves measured in the solar simulator under a 610 nm long pass optical filter.

In order to better understand the observed impact of illumination wavelength on the solar cell parameters, JV curves were taken for both devices in the solar simulator with the inclusion of a 610 nm long pass optical filter between the light source and the cells (Fig. 10). This filter only allows transmission of wavelengths longer than the cut-on wavelength of 610 nm, essentially illuminating the micro-concentrator devices with red light (like the 660 nm laser used for the power series). The long pass filter reduces the *J*_*sc*_ of both devices as expected since the blue part of the spectrum is cut out. For both devices, an increase in *V*_*oc*_ is observed, with S500 gaining about 30 mV, similar in magnitude to the gain of about 60 mV observed for the 660 nm laser illumination. Also, R_shunt_ increases with the introduction of the long pass filter for both devices as also observed during laser illumination. This can be explained by the photoconductivity of CdS which is known to increase under blue light illumination for some devices^48,49^. S500 has a high number of pinholes (Fig. 3c,e) which were filled with CdS during device processing. If now the conductivity of CdS is increased, those filled holes create a direct shunt path between front and back contact. Thus, explaining the higher *V*_*oc*_ under red light illumination.

## Conclusion

An overall analysis of the process of area-selective electrodeposition of a CIGSe absorber layer into micro-patterned substrates, the assembly of micro-concentrator photovoltaic devices and the characterization of the absorber and device are presented in this work. Metallic stacks consisting of copper, indium and gallium were electrodeposited into microelectrodes of 200 µm diameter. Evidence of a change of deposition behaviour between microelectrodes and large area electrodes was observed, as controlling the local and global composition proved to be difficult. Ongoing experimental work focusses on this as the opto-electronic properties of CIGSe crucially depend on the composition. The metallic stacks were annealed at a relatively low temperature of 450 °C to form mostly the chalcopyrite phase, as well as the OVC compound CuIn_5_Se_8_, for Cu-poor compositions. We showed that a stacked metal precursor approach gave higher open circuit voltages compared to the “all elements being deposited at once” approach previously used for these SiO_2_ templates, which is the same result as found for large area electrodeposition. Under one-sun illumination the thinnest absorber layer device collected a similar amount of current as compared to the thickest absorber layer but had a significantly lower open circuit voltage presumably caused by a shunt resistance four times lower than the thickest absorber layer device. Under increased illumination intensity, illumination dependent shunt resistance is identified as the main problem that is limiting device efficiency increase. To improve the performance of these devices, their low shunt resistance and the light dependent shunt resistance must be ameliorated, by making the absorber layers pinhole free, and more tightly control the composition and homogeneity of the absorber layers. Nonetheless, these devices achieve the highest *V*_*oc*_, under light concentration conditions, of a bottom-up micro-CIGSe device published in the literature. Individual micro-concentrator devices showed an increase from 2.2% at 1 × CF to 4.8% at 33 × CF and also from 4.8% at 1 × CF to 5.2% at 3 × CF under 660 nm illumination, clearly demonstrating the potential of area-selective electrodeposition as a material saving technique for the preparation of micro-concentrator solar cells.

## Supplementary information

Supplementary Information
