# Supplementary material for: Micro-sized thin-film solar cells via area-selective electrochemical deposition for concentrator photovoltaics application
Source: Sci Rep. 2020 Sep 8;10:14763. doi: 10.1038/s41598-020-71717-0 (PMC7479101; doi:10.1038/s41598-020-71717-0)
Supplement: Supplementary file 1 — Supplementary Information [file 41598_2020_71717_MOESM1_ESM.docx]

Supporting Information

Table S1: Atomic percentage values determined by EDX. An error of 1% is considered for all values.

| Atomic % | S500 | S1500 |
| --- | --- | --- |
| Cu | 16.9 | 18.9 |
| In | 16.0 | 29.6 |
| Ga | 2.1 | 0.1 |
| Se | 65.0 | 51.4 |


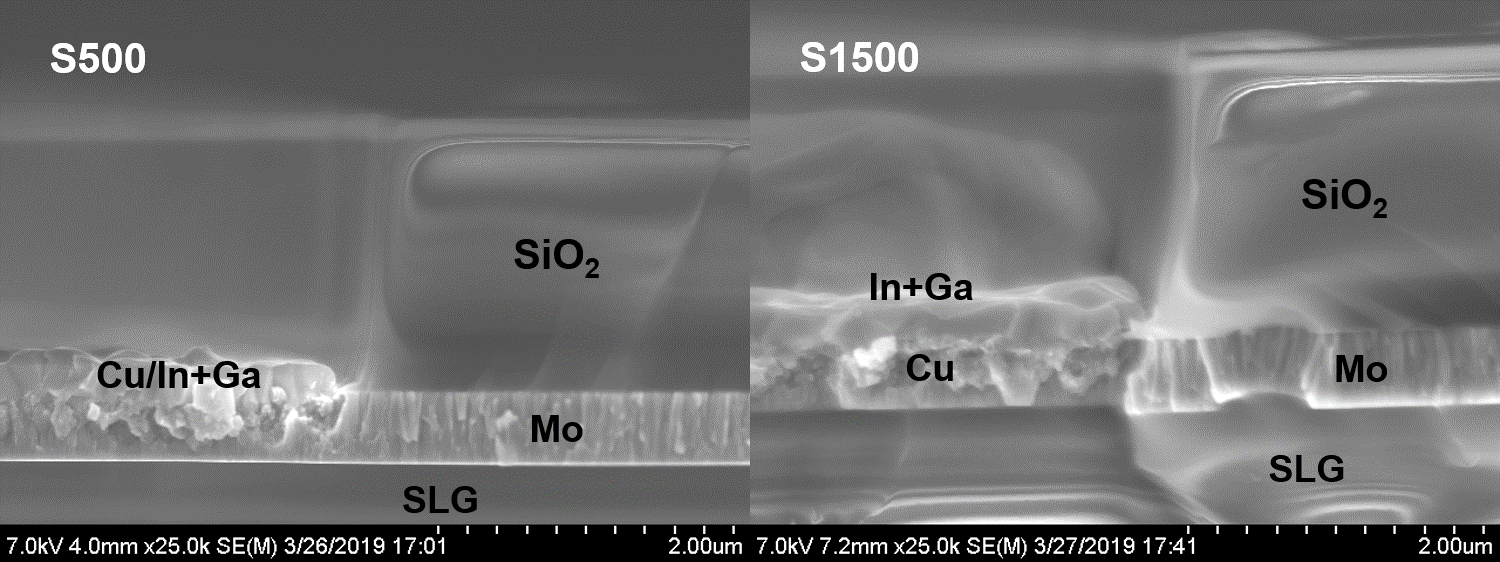


Fig. S1: SEM micrographs of the metallic Cu, In, and Ga precursor layers before selenization. The micrographs show a cross-section of the interface between the SiO_2_ template, the Mo back electrode, and the electrodeposited Cu and In+Ga. Below the Mo layer is a glass substrate. The metallic precursors were prepared in identical conditions to S500 (left) and S1500 (right). In the background of the S1500 sample an indium island may be observed, in agreement with the top view observations in figure 3 of the manuscript.


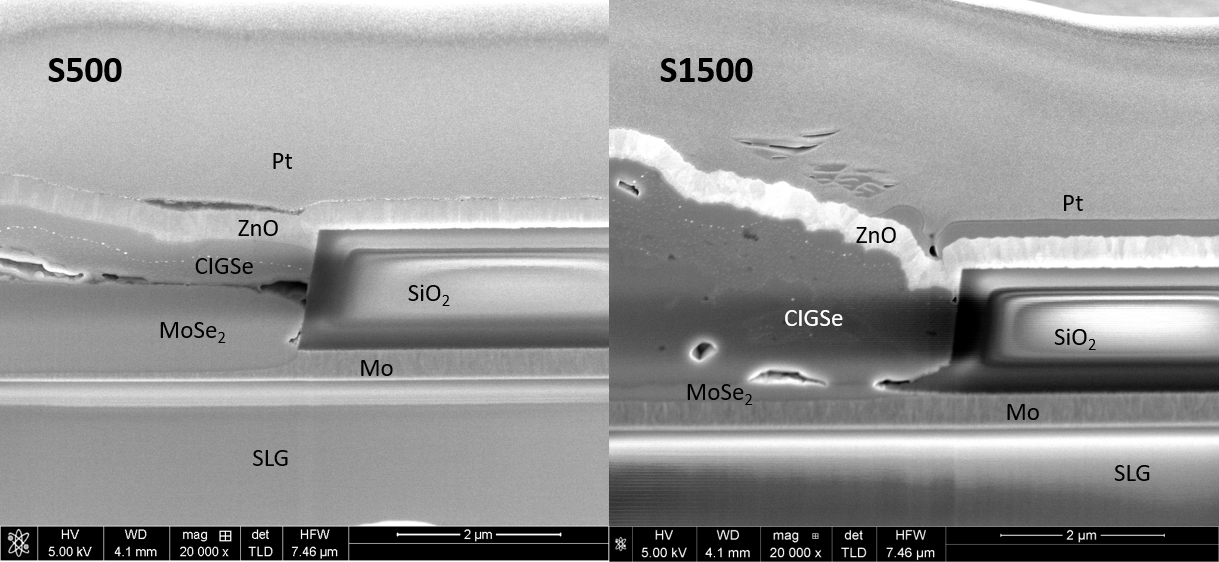


Fig. S2: FIB-SEM lamella micrographs for devices prepared using S500 (left) and S1500 (right) absorber layers, showing a cross-section of the interface between the SiO_2_ template, the Mo back electrode, and the CIGSe absorber layer. In addition, on top of the CIGSe, the ZnO layers of the device can be seen, as well as the Pt layer needed for protection and imaging. Note no direct connection can be observed between the Mo and the ZnO layers at the SiO_2_ wall.


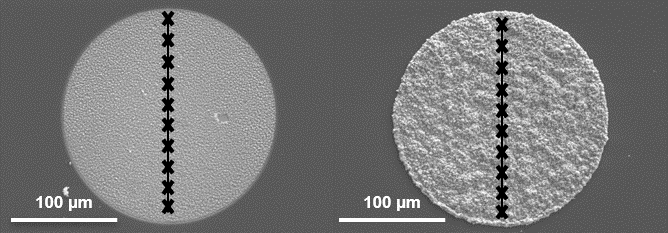


Fig. S3: SEM micrographs of the S500 (left) and the S1500 (right) absorber layers, showing the positions of the scans used to generate the Raman spectra shown in Fig 5. The incident beam size was approximately 1 µm.
